# Rush Medical College

**Published:** 1845-06

**Authors:** 


### RUSH MEDICAL COLLEGE.

The Third Annual Course of Lectures in this Institution will commence on the First Monday of November, 1845, and continue 16 weeks.

The organization of the Faculty is at present as follows:

Surgery and Surgical Anatomy, Daniel Brainard, M.D., of Chicago.

Chemistry and Pharmacy, J. V. Z. Blaney, M.D.

Materia Medica and Therapeutics, John McLean, M.D., of Jackson, Mich.

General and Descriptive Anatomy, Wm. B. Herrick, M.D., of Chicago.

Institutes and Practice of Medicine, G. N. Fitch, M. D., of Logansdort, Indiana.

Obstetrics and Diseases of Women and Children, John Evans, M. D., of Indianapolis, Indiana.

The annual circular will soon appear. The class of last (2d) session numbered 46. Graduates 11. The first class numbered but 22. The fees for the entire course, are $60. Matriculation fee, $5. Graduation fee, $20.

